# ACUTE LOWER RESPIRATORY INFECTION IN GUARANI INDIGENOUS CHILDREN, BRAZIL

**DOI:** 10.1590/1984-0462/;2018;36;2;00017

**Published:** 2018-03-29

**Authors:** Patricia Gomes de Souza, Andrey Moreira Cardoso, Clemax Couto Sant’Anna, Maria de Fátima Bazhuni Pombo March

**Affiliations:** a Universidade Federal do Rio de Janeiro, Rio de Janeiro, RJ, Brasil.; b Fundação Oswaldo Cruz, Rio de Janeiro, RJ, Brasil.

**Keywords:** Respiratory tract infections, Pneumonia, Indigenous population, Child, Infecções respiratórias, Pneumonia, População indígena, Criança

## Abstract

**Objective:**

To describe the clinical profile and treatment of Brazilian Guarani indigenous children aged less than five years hospitalized for acute lower respiratory infection (ALRI), living in villages in the states from Rio de Janeiro to Rio Grande do Sul.

**Methods:**

Of the 234 children, 23 were excluded (incomplete data). The analysis was conducted in 211 children. Data were extracted from charts by a form. Based on record of wheezing and x-ray findings, ALRI was classified as bacterial, viral and viral-bacterial. A bivariate analysis was conducted using multinomial regression.

**Results:**

Median age was 11 months. From the total sample, the ALRI cases were classified as viral (40.8%), bacterial (35.1%) and viral-bacterial (24.1%). It was verified that 53.1% of hospitalizations did not have clinical-radiological-laboratorial evidence to justify them. In the multinomial regression analysis, the comparison of bacterial and viral-bacterial showed the likelihood of having a cough was 3.1 times higher in the former (95%CI 1.11-8.70), whereas having chest retractions was 61.0% lower (OR 0.39, 95%CI 0.16-0.92). Comparing viral with viral-bacterial, the likelihood of being male was 2.2 times higher in the viral (95%CI 1.05-4.69), and of having tachypnea 58.0% lower (OR 0.42, 95%CI 0.19-0.92).

**Conclusions:**

Higher proportion of viral processes was identified, as well as viral-bacterial co-infections. Coughing was a symptom indicative of bacterial infection, whereas chest retractions and tachypnea showed viral-bacterial ALRI. Part of the resolution of non-severe ALRI still occurs at hospital level; therefore, we concluded that health services need to implement their programs in order to improve indigenous primary care.

## RESUMO

**Objetivo:**

Descrever o perfil clínico e o tratamento realizado nas crianças da etnia Guarani menores de cinco anos hospitalizadas por infecção respiratória aguda baixa (IRAB), residentes em aldeias nos estados do Rio de Janeiro ao Rio Grande do Sul.

**Métodos:**

Das 234 crianças, 23 foram excluídas (dados incompletos), sendo analisadas 211. Os dados foram extraídos dos prontuários por meio de formulário. Com base no registro de sibilância e padrão radiológico, a IRAB foi classificada em: bacteriana, viral e viral-bacteriana. Foi utilizada regressão multinomial para análise bivariada.

**Resultados:**

A mediana de idade foi de 11 meses. Do total da amostra, os casos de IRAB foram assim distribuídos: viral (40,8%), bacteriana (35,1%) e viral-bacteriana (24,1%). Verificou-se que 53,1% das hospitalizações não possuíam evidências clínico-radiológico-laboratoriais que as justificassem. Na análise de regressão multinomial, ao comparar a IRAB bacteriana com a viral-bacteriana, a chance de ter tosse foi 3,1 vezes maior na primeira (intervalos de 95% de confiança - IC95% 1,11-8,70) e de ter tiragem 61,0% menor (*Odds Ratio* - OR 0,39, IC95% 0,16-0,92). Na comparação da IRAB viral com a viral-bacteriana, a chance de ser do sexo masculino foi 2,2 vezes maior na viral (IC95% 1,05-4,69) e de ter taquipneia, 58,0% menor (OR 0,42, IC95% 0,19-0,92) na mesma categoria.

**Conclusões:**

Identificou-se maior proporção de processos virais do que processos bacterianos, bem como a presença de infecção viral-bacteriana. A tosse foi um sintoma indicativo de infecção bacteriana, enquanto a tiragem e a taquipneia apontaram infecção viral-bacteriana. Parte da resolubilidade da IRAB não grave ocorreu em âmbito hospitalar; portanto, propõe-se que os serviços priorizem ações que visem à melhoria da assistência à saúde indígena na atenção primária.

## INTRODUCTION

At a global level, acute respiratory infections (ARIs) are the main cause of morbimortality among children. In developing countries, pneumonias - one of the main acute lower respiratory infections (ALRIs)^1^ - are in charge of 20 to 40% of hospitalizations of children aged less than 5 years, and of 20% of deaths in the same age group.^2^

In infant health, ALRIs are mainly represented by community-acquired pneumonia (CAP)^1^ and acute viral bronchiolitis (AVB).^3^ Even though most ALRIs are caused by a virus, the viral-bacterial infection is observed in more than one fourth of the hospitalized children.^4^ In 83 Guarani villages, located in the South and Southeast of Brazil, the proportion of hospitalizations due to the same cause was 77.6% in children aged less than five years, and 83.4% in children aged less than one year.^5^ The high prevalence of wheezing found among Guarani children who were hospitalized by ALRI^6^ suggests high frequency of viral etiology,^3^ as well as of viral-bacterial infection.^7^

In developing countries, because of the difficulties to conduct laboratory tests to identify the viruses,^8^ the clinical-radiological criteria are the most used to distinguish the viral and the bacterial infections.^1^ It is necessary to know the profile of the ALRIs caused by different agents to formulate the treatment and reduce the unnecessary use of antiobiotics.^9^ ^,^ ^10^ This study aimed at describing the clinical profile and the treatment carried out with Guarani children hospitalized because of ALRI in the South and Southeast of Brazil, as well as at gathering evidence to assist the presumption of the etiology and the therapeutic decision-making in contexts with limited diagnostic resources.

## METHOD

A cross-sectional study about the clinical profile and treatment of Guarani indigenous children aged less than five years hospitalized because of ALRI (2007-2008), living in 83 villages in the states of Rio de Janeiro down to Rio Grande do Sul. Indigenous primary health care is provided by a multidisciplinary indigenous health team managed by the Indigenous Health Care Subsystem, component of the Unified Health System (SUS). Hospitalizations are referred to the SUS network outside the villages by the Reference and Counter-Reference System.^11^

The hospitalizations were recruited based on the surveillance system of a case-control study,^12^ and data extraction of the medical charts was conducted by using a standardized form. The methodological details are described in other articles.^5^ ^,^ ^12^

The variables are:

- Demographic and hospitalization seasons of the year: age group (0 to <2 months, 2 to 23 months and 24 to 59 months), sex, region of residence (South or Southeast) and seasons of the year (fall, winter, spring and summer).
- Clinics: hospital complexity in the National Record of Health Institutions, categorized in high and medium, based on the higher level of complexity in the hospital.
- Diagnostic hypothesis (DH) of the assistant physician used for the diagnostic classification of the study, based on the etiological hypotheses: CAP^1^=bacterial ALRI, AVB^3^=viral ALRI, and CAP+AVB = viral-bacterial ALRI.

Signs and symptoms assessed: cough; fever; dyspnea; tachypnea; rales; retractions; wheezes; signs of severity; simultaneous presence of other conditions, such as recurrent wheezing - asthma or wheezing infant, cardiopathy and repetitive pneumonia. The classification of nutritional status was conducted based on “weight/age”, standardized in Z score: adequate weight for age (≥Z score -2 and ≤Z score +2) and low weight for age (<Z score -2). The clinical, laboratory and radiological evidence was used to indicate hospitalization^1^ ^,^ ^3^. The laboratory evidence was based on leukocyte and rod count, anemia (hemoglobin <11 g/dL), peripheral oxygen saturation (SpO_2_) and blood culture. The radiological pattern was classified as:

- Viral: interstitial opacity, atelectasis, lung hyperinflation.
- Bacterial: alveolar opacity, condensation, pleural effusion, pneumatocele.^1^
- No description.

The treatment was assessed as: antibiotics - adequate dose for weight, adequate interval between doses, recommended duration, beginning of antibiotic therapy in relation to hospital admission (up to four hours, after four hours), change of antibiotics during hospital stay -, systemic and/or inhaled corticosteroids, nebulization with bronchodilator, oxygen therapy and adaptation of treatment.^1^ ^,^ ^3^ The following variables were also assessed: time of hospitalization (1-3 days, 4-6 days, 7-14 days and 15 days or more), re-hospitalization of the child because of the same episode (up to 14 days after discharge), complications and death. The variables with no record in the chart were defined as absent.

Based on the clinical-radiological criteria of the protocols,^1^ ^,^ ^3^ the diagnostic classification of the study was established as follows:

- Bacterial ALRI: no record of wheezing and thoracic X-ray with at least one of the following changes: condensation, alveolar infiltrate, pleural effusion, pneumatocele, without description of the report or that the examination was not taken.
- Viral ALRI: with record of wheezing and thoracic X-ray with at least one of the following findings: interstitial infiltrate, atelectasis, lung hyperinflation, rectification of costal arches, without description of the report or that the examination was not taken. Viral ALRI is also considered if there is no record of wheezing and thoracic X-ray with at least one of the findings: interstitial infiltrate, atelectasis, lung hyperinflation or rectification of costal arches.
- Viral-bacterial ALRI: with record of wheezing and thoracic X-ray, with at least one of the findings: condensation, alveolar infiltrate, pleural effusion or pneumatocele.

The indication of hospitalization was assessed considering age, clinical-radiological aspects and saturation of oxygen corresponding to the hospitalization criteria of CAP and AVB.^1^ ^,^ ^3^

The adequation of the treatment was analyzed based on the assistant physician’s DH, following the recommendations of the protocols.^1^ ^,^ ^3^ The indication of the following medications was assessed: prescription of antibiotics, use of empiric antibiotics as the first choice, oxygen therapy, nebulization with bronchodilator and corticotherapy.

The data base and the statistical analysis were conducted using the software SPSS Statistics, version 21. In the descriptive analysis, mean±standard deviation (SD) and median were calculated for the continuous variables, and distribution of absolute and relative frequencies was used for categorical variables. The diagnostic classification of the study was considered as a dependent variable. The following step was the analysis of the distribution of variables of interest according to the categories of diagnostic classification of the study, using the chi-square test or Fisher’s exact test to verify the statistically significant differences in the distributions, considering a 5% significance level, besides the calculation of the respective 95% confidence interval (95%CI) values.

Afterwards, there was a bivariate analysis using the multinomial logistic regression, estimating, with the Odds Ratio (OR), the association between variables, considering the viral-bacterial ALRI as the category of reference.

This sub-project is part of a case-control study about acute respiratory conditions affecting Guarani indigenous people.^12^ The general project was submitted to and approved by the Research Ethics Committee of National Public Health School at Fundação Oswaldo Cruz (Fiocruz) and by the National Research Ethics Commission.

## RESULTS

This study included 234 children with ALRI, of which 23 were excluded due to incomplete data regarding clinical management. The analysis was carried out with 211 children, with mean age of 11 months (zero to 58 months), and 75% of them were aged less than 21 months.

Of the total, 86 (40.8%) were classified with viral ALRI, 74 (35.1%), with bacterial ALRI, and 51 (24.1%), with viral-bacterial ALRI. There was higher frequency of hospitalization among female participants (51.7%), those aged less than 24 months (79.6%), living in the Southeast (67.3%), in high complexity hospitals (70.1%), and in autumn (34.2%), as observed in Table 1. Gender was significantly associated with ALRI, and a higher proportion of boys presented with viral ALRI. The other variables were not associated with the type ot ALRI (Table 1).

**Table 1:** Frequency of hospitalization and proportion of acute lower respiratory infection, according to demographic and climactic variables.

	Hospitalization due to ALRI		Bacterial ALRI			Viral ALRI			Viral-bacterial ALRI			p-value*
	n	%	n	%	95%CI	n	%	95%CI	n	%	95%CI	
Sex												
Male	102	48.3	29	39.2	28.6-50.6	51	59.3	48.7-69.3	22	43.1	30.1-56.9	0.028
Female	109	51.7	45	60.8	49.4-71.4	35	40.7	30.7-51.3	29	56.9	43.1-69.9	
Age (months)												
0 to <2	14	6.6	6	8.1	3.4-16.1	7	8.1	3.6-15.4	1	2.0	0.1-9.3	0.290
2 to 23	154	73.0	49	66.2	54.9-76.3	63	73.3	63.2-81.8	42	82.3	70.1-91.0	
24 to 59	43	20.4	19	25.7	16.7-36.5	16	18.6	11.4-27.9	8	15.7	7.6-27.6	
Region of residence												
South	69	32.7	19	25.7	16.7-36.5	32	37.2	27.5-47.8	18	35.3	23.2-49.1	0.271
Southeast	142	67.3	55	74.3	63.5-83.3	54	62.8	52.2-72.5	33	64.7	50.9-76.9	
Season of hospitalization												
Autumn	72	34.2	25	33.8	23.7-45.1	27	31.4	22.3-41.8	20	39.2	26.6-53.0	0.903
Winter	54	25.6	21	28.4	19.0-39.4	23	26.7	18.2-36.8	10	19.6	10.4-32.2	
Spring	60	28.4	19	25.7	16.7-36.5	25	29.1	20.2-39.3	16	31.4	19.8-45.0	
Summer	25	11.8	9	12.2	6.1-21.1	11	12.8	6.9-21.1	5	9.8	3.7-20.4	
Hospital complexity												
High	148	70.1	48	64.9	53.5-75.1	60	69.8	59.5-78.8	40	78.4	65.6-88.1	0.264
Medium	63	29.9	26	35.1	24.9-46.5	26	30.2	21.2-40.5	11	21.6	11.9-34.4	

*ALRI: lower acute respiratory infection; Chi-square test; 95%CI: 95% confidence interval.

CAP (167; 79.2%) was the most important cause of hospitalization. The diagnostic classification of the study was not compatible with the assistant physician’s DH in 54.5% of the cases; 53.1% of the hospitalizations did not have clinical, radiological or laboratory evidence to justify them (Table 2).

**Table 2:** Frequency of hospitalization and proportion of acute lower respiratory infection, according to clinical variables.

	Hospitalization due to ALRI		Bacterial ALRI			Viral ALRI			Viral-bacterial ALRI			p-value*
	n	%	n	%	95%CI	n	%	95%CI	n	%	95%CI	
APDH compatible with SC												
Yes	96	45.5	71	95.9	89.4-99.0	21	24.4	16.2-34.3	4	7.8	2.5-17.8	<0.001
No	115	54.5	3	4.1	1.0-10.6	65	75.6	65.7-83.8	47	92.2	82.2-97.5	
Evidence for hospitalization												
Yes	99	46.9	30	40.5	29.8-52.0	43	50.0	39.5-60.5	26	51.0	37.4-64.5	0.392
No	112	53.1	44	59.5	48.0-70.2	43	50.0	39.5-60.5	25	49.0	35.6-62.6	
Presence of comorbidity												
Yes	92	43.6	30	40.5	29.8-52.0	31	36.0	26.4-46.6	31	60.8	47.0-73.4	0.015
No	119	56.4	44	59.5	48.0-70.2	55	64.0	53.4-73.6	20	39.2	26.6-53.0	
Weight/age												
Adequate	66	37.5	21	37.5	25.6-50.7	29	38.7	28.2-50.0	16	35.6	22.7-50.2	0.919
Risk	60	34.1	17	30.4	19.4-43.3	27	36.0	25.8-47.3	16	35.6	22.7-50.2	
Low weight	50	28.4	18	32.1	20.9-45.2	19	25.3	16.5-36.1	13	28.9	17.1-43.3	
Tachypnea												
Yes	110	52.1	34	45.9	34.9-57.4	41	47.7	37.3-58.2	35	68.6	55.0-80.2	0.025
No	101	47.9	40	54.1	42.7-65.1	45	52.3	41.8-62.7	16	31.4	19.8-45.0	
Dyspnea												
Yes	136	64.5	41	55.4	44.0-66.4	57	66.3	55.8-75.7	38	74.5	61.3-85.0	0.081
No	75	35.5	33	44.6	33.6-56.0	29	33.7	24.3-44.2	13	25.5	15.0-38.7	
Retraction												
Yes	65	30.8	14	18.9	11.2-29.0	31	36.0	26.4-46.6	20	39.2	26.6-53.0	0.021
No	146	69.2	60	81.1	71.0-88.8	55	64.0	53.4-73.6	31	60.8	47.0-73.4	
Fever												
Yes	141	66.8	54	73.0	62.0-82.2	55	64.0	53.4-73.6	32	62.7	48.9-75.1	0.374
No	70	33.2	20	27.0	17.9-38.0	31	36.0	26.4-46.6	19	37.3	24.9-51.1	
Cough												
Yes	169	80.1	65	87.8	78.9-93.9	69	80.2	70.8-87.6	35	68.6	55.0-80.2	0.030
No	42	19.9	9	12.2	6.1-21.1	17	19.8	12.4-29.2	16	31.4	19.8-45.0	
Rales												
Yes	144	68.2	47	63.5	52.1-73.9	56	65.1	54.6-74.6	41	80.4	67.8-89.6	0.099
No	67	31.8	27	36.5	26.1-47.9	30	34.9	25.4-45.4	10	19.6	10.4-32.2	

*ALRI: acute lower respiratory infection; chi-square test; APDH: assistant physician’s diagnostic hypothesis; SC: study classification; Risk: nutritional risk; 95%CI: 95% confidence interval.

There was a record of comorbidity in 43.6% of the hospitalizations, mostly due to recurrent wheezing conditions. The adequate weight for age was verified in 71.6% of the 176 children with record of weight. Tachypnea, dyspnea, retraction, fever, cough and rales presented frequencies ranging from 30.8% (retraction) to 80.1% (cough), as observed in Table 2, whereas wheezing was observed in 125 (59.2%) charts (data not tabulated). There was no seizure, sleepiness and grinding; The frequency of refusal to eat or drink was lower than 5%, so these data were excluded from the analysis.

ALRI was associated with the compatibility between the classification of the study and the physician’s DH, comorbidities, tachypnea, retraction and cough. The presence of compatibility and cough was more common in bacterial ALRI, whereas retraction was less common in this group. Besides, the record of comorbidities and tachypnea was higher in viral-bacterial ALRI (Table 2).

Of the analyzed charts, there was absence of 26 (12.3%) records of blood counts, 16 (7.6%) thoracic X-rays, 187 (88.6%) blood cultures and 186 (88.2$) records of SpO_2_. The analyses excluded blood culture and SpO_2_, because of the low frequency of the records. The frequency of the alveolar infiltrate associated or not with pleural effusion and/or pneumatocele was 48.2%, interstitial infiltrate associated or not with atelectasis and/or lung hyperinflation and/or rectification of costal arches was 20.5%, whereas 31.3% of the X-rays did not have reports. Of the 25 records of SpO_2_, 13 presented with hypoxemia. Two blood cultures were positive for *Staphylococcus aureus* - in both cases, it was the bacterial ALRI. Leukocytosis with left shift was registered in 50.8% of the children, and anemia, in 68.1% of the cases, however, without association with ALRI.

Antibiotics were prescribed in 206 (97.6%) hospitalizations, of which 20 (9.7%) had the physician’s DH of AVB. It was observed that 147 (71.4%) of the children started on antibiotic therapy in the first four hours after admission. Penicillin was the most used empiric antibiotics as the first choice (85.9%), followed by cephalosporins (12.1%). The prescribed dose of the antibiotic was considered adequate for the child’s weight in 52.3% of the hospitalizations (Table 3). The intravenous path was the most used one (86.4%), and the interval between doses was considered adequate in 94.2% of the prescriptions. The change of the first antibiotic took place in half of the hospitalizations, in average every 3.3 days. Also regarding the treatment, 201 (95.3%) patients used nebulization with bronchodilator; 24 (11.4%), inhaled corticosteroid; 27 (12.8%), oxygen therapy; and 145 (68.7%), systemic corticosteroid. A little more than one third of the children was offered treatment considered to be adequate based on the protocols.

**Table 3:** Frequency of hospitalization and proportion of acute lower respiratory infection, according to treatment and outcome.

	Hospitalization due to ALRI		Bacterial ALRI			Viral ALRI			Viral-bacterial ALRI			p-value*
	n	%	n	%	95%CI	n	%	95%CI	n	%	95%CI	
ATB dose adequate for weight												
Yes	90	52.3	30	52.6	39.7-65.3	36	50.7	39.2-62.2	24	54.5	39.8-68.7	0.921
No	82	47.7	27	47.4	34.7-60.3	35	49.3	37.8-60.8	20	45.5	31.3-60.2	
Beginning of ATB in hospital												
Up to 4 hours	147	71.4	63	85.1	75.6-91.9	52	63.4	52.6-73.3	32	64.0	50.1-76.4	0.005
After 4 hours	59	28.6	11	14.9	8.1-24.4	30	36.6	26.7-47.4	18	36.0	23.7-49.9	
Adequate interval of ATB doses												
Yes	194	94.2	72	97.3	91.4-99.5	77	93.9	87.0-97.7	45	90.0	79.2-96.2	0.222
No	12	5.8	2	2.7	0.5-8.6	5	6.1	2.3-13.0	5	10.0	3.8-20.8	
Adequate duration of ATB												
Yes	57	27.7	24	32.4	22.5-43.7	21	25.6	17.1-35.9	12	24.0	3.7-37.2	0.509
No	149	72.3	50	67.6	56.3-77.5	61	74.4	64.1-83.0	38	76.0	62.8-86.3	
Change of ATB												
Yes	103	50.0	33	44.6	33.6-56.0	37	45.1	34.6-56.0	33	66.0	52.1-78.1	0.034
No	103	50.0	41	55.4	44.0-66.4	45	54.9	44.0-65.4	17	34.0	21.9-47.9	
Use of corticosteroid												
Yes	145	68.7	36	48.6	37.4-60.0	68	79.1	69.5-86.7	41	80.4	67.8-89.6	<0.001
No	66	31.3	38	51.4	40.0-62.6	18	20.9	13.3-30.5	10	19.6	10.4-32.2	
Adequate treatment												
Yes	79	37.4	21	28.4	19.0-39.4	27	31.4	22.3-41.8	31	60.8	47.0-73.4	<0.001
No	132	62.6	53	71.6	60.6-81.0	59	68.6	58.2-77.7	20	39.2	26.6-53.0	
Hospitalization days												
1 to 3	29	13.7	9	12.2	6.1-21.1	17	19.8	12.4-29.2	3	5.9	1.5-15.2	0.167
4 to 6	87	41.3	33	44.6	33.6-56.0	36	41.8	31.8-52.5	18	35.3	23.2-49.1	
7 to 14	76	36.0	25	33.7	23.7-45.1	28	32.6	23.3-43.0	23	45.1	31.9-58.8	
15 or more	19	9.0	7	9.5	4.2-17.8	5	5.8	2.2-12.4	7	13.7	6.2-25.3	

*ALRI: acute lower respiratory infection; Chi-square test or Fisher’s exact test; ATB: antibiotic; 95%CI: 95% confidence interval.

The beginning of the antibiotic, its change, the corticosteroid and the adaptation to treatment were associated with ALRI. More patients with bacterial ALRI started on antibiotics up to four hours after hospitalization, whereas the use of corticosteroid was lower in this group. On the other hand, the fact of changing antibiotics and having adequate treatment was more frequent in viral-bacterial ALRI (Table 3).

The mean time of hospital stay was 7.8±6.6 days, ranging from one to 63 days, considering that 8.5% of the hospitalizations lasted for less than three days, and 79.1%, for up to ten (Table 3). Eight children were re-hospitalized because of the same episode, two were transferred for intensive care, and one died (classified as viral-bacterial ALRI).

In the multinomial regression analysis, by comparing bacterial ALRI with the viral-bacterial ALRI, in the former the chances of cough were 3.1 times higher (95%CI 1.11-8.70), and retraction, 61.0% lower (OR 0.39, 95%CI 0.16-0.92). In the comparison between viral ALRI and viral-bacterial ALRI, in the former the chances of being male was 2.2 times higher (95%CI 1.05-4.69) and of having tachypnea, 58.0% lower (OR 0.42, 95%CI 0.19-0.92), as observed in Table 4.

**Table 4:** Acute lower respiratory infection and associated factors, according to the multinomial logistic regression.

	Bacterial ALRI		Viral ALRI	
	OR (95%CI)	p-value	OR (95%CI)	p-value
Sex				
Female	1.00	0.77	1.00	0.04
Male	0.89 (0.40-1.96)		2.22 (1.05-4.69)	
Tachypnea				
No	1.00	0.12	1.00	0.03
Yes	0.52 (0.23-1.18)		0.42 (0.19-0.92)	
Dyspnea				
No	1.00	0.16	1.00	0.59
Yes	0.53 (0.22-1.27)		0.79 (0.34-1.85)	
Retraction				
No	1.00	0.03	1.00	0.99
Yes	0.39 (0.16-0.92)		1.02 (0.46-2.16)	
Fever				
No	1.00	0.52	1.00	0.63
Yes	1.34 (0.55-3.29)		0.81 (0.35-1.88)	
Cough				
No	1.00	0.03	1.00	0.12
Yes	3.10 (1.11-8.70)		2.05 (0.82-5.11)	
Rales				
No	1.00	0.13	1.00	0.13
Yes	0.51 (0.21-1.23)		0.52 (0.22-1.22)	

Category of reference: viral-bacterial ALRI; ALRI: acute lower respiratory infection; OR: *Odds Ratio*; 95%CI: 95% confidence interval.

## DISCUSSION

The disparity between the health of indigenous and non-indigenous peoples is clear,^13^ ^,^ ^14^ ^,^ ^15^ and this has been attributed, for instance, to the poor socioeconomic conditions, the high load of infectious diseases, and the limitations in the continuity of their traditional means of subsistence.^16^

This study identified a higher proportion of presumed viral processes, and it points to the presence of viral-bacterial coinfection. This seems to represent higher severity and worse therapeutic response, whereas the cases of non-combined viral or bacterial ALRI present a challenge to diagnosis and to the decision regarding the therapy. The study contributes with the subject not only because of the knowledge of the clinical profile manifested by Guarani children hospitalized with ALRI, but especially for its proposal of a clinical-radiological classification of the etiological hypothesis.

ALRI is one of the main causes of hospitalization of indigenous children in developed^7^ ^,^ ^17^ and developing^5^ countries. In the case of the Guarani children, our results indicate high frequency of hospitalization for non-severe ALRI (53.1%), with slight prevalence of viral ALRI in comparison to bacterial ALRI. Once these conditions are potentially treatable in primary health care,^1^ ^,^ ^3^ we observed that the indication for hospitalization is not always clinical, but it may result from factors such as the difficulty of the family regarding home care,^1^ language limitations, difficulties to schedule appointments and to obtain medication, and the high turnover of professionals, as well as their insufficient skills to manage these cases.^5^

There were more hospitalizations caused by viral ALRI in male children aged less than 24 months, which is similar to studies conducted with indigenous populations from New Zealand^18^ and the United States,^19^ ^,^ ^20^ as well as non-indigenous children.^21^ According to the literature, the household agglomeration and the exposure to the wood burning stove smoke were associated with higher risk of hospitalization due to AVB in indigenous children.^12^

Even though most ALRIs are viral,^18^ viral-bacterial infections were observed in approximately one fourth of the non-indigenous hospitalized children,^22^ ^,^ ^23^ as in this study, in which the number represented 24.1%. Studies with indigenous peoples in Australia^7^ reported the occurrence of viral-bacterial coinfection in patients with ALRI.

Studies of CAP etiology in a non-indigenous population reported frequency of viral-bacterial infection in 30 to 66% of the cases.^22^ ^,^ ^24^ In the coinfections, it is common for the *S. pneumoniae* to be associated with the human rhinovirus^10^ ^,^ ^23^ ^,^ ^25^ or to the respiratory syncytial virus.^10^ ^,^ ^23^ In the cases reported here, this analysis was not conducted due to the absence of examinations for the research of respiratory viruses, besides the low request for blood culture.

Cough, retraction and tachypnea were associated with ALRI, confirming their importance for diagnosis.^1^ ^,^ ^3^ Cough was associated with bacterial ARLI, demonstrating to be a symptom that indicates bacterial infection,^1^ whereas retraction and tachypnea were associated with viral-bacterial ALRI. Once retraction and tachypnea are also present in bacterial ALRI,^1^ the valorization of these clinical data added to wheezing would help the clinical differential diagnosis between bacterial and viral-bacterial ALRI.^1^ ^,^ ^3^

Thoracic X-ray, blood test, blood culture and SpO_2_ are recommended for children with CAP who need hospitalization.^1^ Even though almost 80% of the hospitalizations were caused by CAP, it was observed that not all patients underwent these tests. This fact can reflect the operational difficulties in the health units, or the insufficient knowledge about the protocols.^1^ The radiological pattern was mostly showing alveolar infiltrate, associated or not with pleural effusion and/or pneumatocele. Considering the high frequency of wheezing in this population, suggesting the existence of bronchial obstruction and the possibility of these cases evolving to atelectasis, there is the possibility of radiological diagnostic error between the alveolar and the interstitial infiltrate and/or atelectasis, overestimating the diagnosis of CAP.^1^

Children with suspicion of viral infection were on antibiotics, nebulization with bronchodilator and corticosteroids in a little discerning manner. Antibiotics and corticosteroids are not recommended for AVB, and the use of bronchodilators is controversial, used as therapeutic evidence and maintained in the presence of clinical response.^3^ This conduct may be attributed to the difficulty to distinguish bacterial and viral ALRI^1^ ^,^ ^26^ and to the absence of a routine medical team. The last hypothesis could explain the change of empiric antibiotics in half of the hospitalizations, despite its apparent initial adaptation.

The mean time of hospitalization was longer than in non-indigenous children, in accordance with some studies,^27^ which leads to the conclusion that, in the absence of clinical severity^1^ for hospital discharge, other factors should be taken into consideration. It is the case of malnutrition, which may lead to reduced immunity; living in indigenous areas of difficult access; lack of public vehicles addressed to the transportation of patients and lack of structure in primary health care to continue the treatment.^28^

There were limitations inherent to a study based on secondary data, such as incomplete records in charts, which prevented some analyses. This fact shows the need to improve the hospital records, in order to improve care and the offer of information about the use and quality of services, thus contributing with the evaluation of health policies, medical-care strategies and research.^29^ Besides, the classification of ALRI used in this study may be criticized, once it was carried out in an arbitrated manner, without the proper etiological proof of the cases.

This study suggests that part of the resolution of non-severe ALRI cases in Guarani children happens in the hospital environment. This model is opposed to the guidelines of the National Policy of Indigenous Peoples Health Care^11^ and the National Policy of Children’s Health Care;^30^ both are based on integral health care, guided by principles that aim not only at curing diseases, but also at prevention and health promotion for children.

The proposal is that services addressed to these populations prioritize actions whose goal is to improve primary health care in the villages, reducing hospitalization rates. On the other hand, in the hospital environment, there is the need to create medical routines followed by continuous professional training, with good articulation between the levels of care, through an efficient reference and counter-reference system.

Besides, it is important to conduct studies that aim at analyzing the identification of causal agents of ALRIs in indigenous populations, in order to subsidize adequate programs in child care.
